# Management of Acute Myeloid Leukemia in Older Patients: An Updated Canadian Consensus

**DOI:** 10.3390/curroncol33070429

**Published:** 2026-07-18

**Authors:** Andre C. Schuh, Nicholas Chornenki, Mohamed A. Elemary, Mahmoud Elsawy, Brett L. Houston, Brian Leber, Lee Mozessohn, Mitchell Sabloff, Brittany Salter, David Sanford, Joseph Brandwein

**Affiliations:** 1 Departments of Medical Oncology and Hematology, Princess Margaret Cancer Centre, University Health Network, Toronto, ON M5G 2M9, Canada; 2 Department of Medicine, University of Toronto, Toronto, ON M5S 1A8, Canada; 3 Division of Hematology, University of British Columbia, Vancouver, BC V5Z 1M9, Canada; 4 Institute of Health Policy, Management and Evaluation Toronto, University of Toronto, ON M5T 3M6, Canada; 5 Sunnybrook Odette Cancer Centre, Toronto, ON M4N 3M5, Canada; 6 Saskatoon Cancer Centre, Saskatoon, SK S7N 4H4, Canada; 7 Division of Oncology, College of Medicine, University of Saskatchewan, Hematology, Saskatoon, SK S7N 5E5, Canada; 8 Division of Hematology and Hematologic Oncology, Department of Medicine, Dalhousie University, Halifax, NS B3H 2Y9, Canada; 9 QEII Health Sciences Centre, Halifax, NS B3H 3A7, Canada; 10 Department of Internal Medicine, Max Rady College of Medicine, University of Manitoba, Winnipeg, MB R3E 3P5, Canada; 11 Department of Medical Oncology and Hematology, CancerCare Manitoba, Winnipeg, MB R3E 0V9, Canada; 12 Department of Medicine, McMaster University, Hamilton, ON L8S 4L8, Canada; 13 Division of Hematology, Department of Medicine, University of Ottawa, Ottawa, ON K1H 8M5, Canada; 14 The Ottawa Hospital Research Institute, Ottawa, ON K1H 8L6, Canada; 15 Department of Oncology, McMaster University, Hamilton, ON L8S 4L8, Canada; 16 Division of Hematology, Department of Medicine, University of Alberta, Edmonton, AB T6G 2G3, Canada

**Keywords:** acute myeloid leukemia, older patients, fitness assessment, less-intensive treatment, venetoclax, ivosidenib

## Abstract

These are the third Canadian consensus guidelines on the management of acute myeloid leukemia (AML) in older patients. The first was published in 2013, and the second in 2017. The management of AML in older patients changed significantly over this time span, with decreasing emphasis on the use of intensive chemotherapy and the adoption of new standards of care based on less-intensive therapies: first azacitidine + venetoclax combinations, followed by azacitidine + ivosidenib. Increased use of these new therapies in older patients has raised questions about their use, including how to determine patient suitability, select the most effective therapy, and manage dosing and toxicity. In this third Canadian guideline, we address these questions to provide clarity around these new standards of care and improve clinician understanding and confidence in using them. As more new targeted agents become available and hypomethylating agent-based triplet combinations are used more widely, we fully anticipate that we will, in some years’ time, write a fourth guideline on the management of AML in older patients.

## Simple Summary

These are the third Canadian consensus guidelines on the management of AML in older patients since 2013. In that time, the use of intensive chemotherapy in older patients has diminished significantly and older patients today are far more likely to be treated with new, less-intensive therapies. AML outcomes have improved dramatically as well as patients’ quality of life, as the less-intensive agents are less toxic. As is the case whenever new therapies are introduced, there are questions around the use and safety of the new drugs. In this guideline, we address these questions to provide clarity around the less-intensive therapies and to improve clinician understanding and confidence in using them.

## 1. Introduction

The first Canadian consensus guidelines on the treatment of acute myeloid leukemia (AML) in older patients were published in 2013, at a time when low-dose cytarabine (LDAC) was used more commonly, and the use of hypomethylating agents (HMAs) in AML was in its infancy [1]. Revised guidelines were published in 2017, by which time HMA use was more routine [2]. Since that time, there have been many changes in the treatment of AML in older patients. From a treatment point of view, in parallel with a decreasing emphasis on intensive chemotherapy in older patients, azacitidine + venetoclax and LDAC + venetoclax combinations have become standards of care since key publications in 2020 [3,4], followed by azacitidine + ivosidenib in 2022 [5].

The growing use over the last few years of less-intensive rather than intensive AML therapy in older patients has raised several new questions. First, how does one best assess patients to determine their suitability for intensive versus less-intensive therapy? Second, with the rapid evolution of less-intensive AML treatment options, how does one best choose the appropriate therapeutic approach? And third, as new treatment approaches often lead to management confusion, how does one best deal with adverse-event and dosing issues in less-intensively treated older patients with AML?

This third iteration of Canadian consensus guidelines on treatment of AML in older patients focuses on these three questions, and as AML treatment in older patients continues to evolve, these guidelines are followed by an accompanying article that addresses ongoing elderly AML management controversies that await resolution.

### 1.1. Canadian Leukemia Study Group/Groupe canadien d’étude sur la leucémie (CLSG/GCEL)

The management of acute myeloid leukemia (AML) in older patients remains very heterogeneous in Canada: there are differences in diagnostic and treatment standards and principles, including diverse philosophies of care, differences in age cutoffs for treatment, and variable access to diagnostic tests and new therapies [6,7]. The CLSG/GCEL was established in 2019 with the stated goals of improving the diagnosis and management of leukemia in Canada by collaboratively defining diagnostic and management best practices, promoting national standards of care, fostering leukemia research, and improving new drug access.

This update of Canadian consensus guidelines on the management of AML in older patients was undertaken by the CLSG/GCEL to provide treatment guidance in light of new genetic and cytogenetic information and multiple new therapy options. This is also one of two CLSG/GCEL articles in this issue of *Current Oncology*. The other focuses on controversies in the management of AML in older patients: emerging data and topics on which there is not yet universal consensus. These include risk stratification in patients receiving less-intensive therapy; up-front treatment selection based on specific biology (‘good risk’ mutations, “high-risk” *FLT3*-ITD and *TP53* mutations) and bridge to transplant.

### 1.2. Methods

A panel of CLSG/GCEL members with expertise in the clinical management of AML was convened to develop this consensus statement. The panel met twice virtually to identify key issues with respect to the management of AML in older patients. Relevant literature was identified by panel members, who also provided insights based on personal and institutional experience and judgment. No formal evaluation of evidence was conducted.

Panel members then worked individually and in smaller groups to develop outlines, and subsequently draft text and recommendations for sections of the document. These materials were combined into a consensus document that was reviewed by panel members, who were asked to provide their support for the recommendations, with an opportunity for dissenting opinions to be noted in this document.

The CLSG/GCEL will post this consensus statement on its website (https://clsg.ca) following publication, with the intention of updating iteratively sections of the document as new data become available. AML management is a rapidly evolving area. Online updates by section will allow these recommendations to remain as current as possible over time.

## 2. Assessment of Fitness and Suitability for Therapy

The treatment of AML is complex, and due to the nature of available therapies, patients must be assessed carefully to optimize outcomes, while minimizing morbidity and mortality. Over the past few decades, AML treatment has evolved from initial intensive chemotherapy only, primarily directed to younger, more fit patients, to a growing spectrum of less-intensive therapies that are more widely appropriate for different levels of fitness, including older/frailer patient groups not suitable for intensive approaches. Over time, it has also been recognized that we can assess the eligibility of patients for such therapies with a variety of tools and metrics that assess considerably more than just age. Although these tools have been evaluated in a number of studies, they have not yet been incorporated into routine clinical practice for a variety of reasons that are discussed below.

### 2.1. Metrics for Fitness Assessment

There has to date been no agreement on an ‘optimal’ mechanism or tool to assess fitness in patients with AML. The 2025 European LeukemiaNet (ELN) guideline on fitness assessment strongly endorsed that age alone is not sufficient to define fitness [8]. This view reflects a growing consensus on the difference between chronological and biological age, and that the former is insufficient as a basis for treatment decisions [9]. Indeed, no single metric has been identified as optimal for the comprehensive fitness evaluation of patients with AML [8]. Evaluation of fitness using tools such as the Eastern Cooperative Oncology Group (ECOG) Performance Status Scale [10] and the Rockwood Clinical Frailty Index [11] provide crude descriptive information, but are limited primarily to the domains of physical performance. Importantly, they also do not offer insight on the degree to which poor fitness may compromise or be modulated by effective therapy. By extension, therefore, another important consideration is whether fitness and co-morbidities/biochemical abnormalities at the time of AML diagnosis pre-date the development of leukemia (and thus are likely irreversible), or whether they reflect the development of disease and its acute presentation, and thus may resolve with appropriate leukemia treatment.

A comprehensive geriatric assessment (CGA) is a more extensive multimodal evaluation that also assesses cognition, psychological health, and social supports. The International Society of Geriatric Oncology recommends a CGA prior to the initiation of systemic therapy for older adults [12,13]. While providing a more wide-ranging and in-depth evaluation, CGAs have limitations: they are resource-intensive and there is no generally accepted standard for CGAs in AML. A variety of CGA tools have been developed and reported [12,14,15,16,17,18]. The variability of potential components can be a challenge for practitioners and can also decrease the standardization of assessment and comparisons across institutions. A further criticism of CGAs is that precise cutoffs for definitions of patients who are fit, unfit, or frail, are not well-defined, nor is the relationship between potential cutoffs and AML treatment decisions [19,20]. While there are insufficient data to select one tool over others, we suggest assessing the fitness of AML patients in as many domains as possible to identify areas of potential concern, aid in treatment decision-making, and assess for possible early interventions.

### 2.2. Intensive vs. Less-Intensive Therapy Considerations with Respect to Fitness and Frailty

From the perspective of AML treatment, the evaluation of fitness/frailty most crucially occurs at the time of initial treatment. The characterization of individual patient fitness must be made in the context of both treatment intensity and disease biology, balancing risks vs. benefits. For example, a 75-year-old patient with core-binding factor AML is much more likely to benefit from intensive chemotherapy than a 68-year-old patient with ELN 2022 adverse risk disease who requires allogeneic stem cell transplantation (alloSCT) for curative potential [21]. Recent data suggest that, for the latter patients, less-intensive therapy with azacitidine + venetoclax may be at least as effective in getting patients to transplant, with less toxicity, ref. [22] as described below. Patients with *TP53*-mutated AML have dismal overall survival rates whether they receive intensive chemotherapy, HMA + venetoclax, or HMA alone [23,24]. For such older patients, less-intensive therapies are generally preferred, even if they are more fit.

Ideally, the assessment of fitness should be integrated into the selection of AML treatment. A recent phase II study used a CGA combined with disease biology to allocate AML patients ≥ 60 years of age to intensive or less-intensive therapy [25]. Most patients were allocated to less-intensive therapies, and the 90-day mortality rate with CGA-guided treatment was almost half the mortality rate of historical controls (22% vs. 40%). While clinical trials often include a minimum performance status for enrollment (e.g., ECOG ≤ 2), comprehensive fitness assessments have not been routinely incorporated in prospective therapeutic AML clinical trials.

PARADIGM, an open-label phase II randomized controlled trial (RCT), compared conventional intensive chemotherapy (7 + 3 or CPX-351) to azacitidine + venetoclax in intensive chemotherapy-eligible patients ≥ 18 years of age [22]. Most patients were ELN 2022 adverse or intermediate risk (72% and 15%, respectively). Those with core-binding factor AML or fms-related tyrosine kinase 3 (*FLT3)* mutations were excluded, as were patients < age 60 with nucleophosmin (*NPM1*) mutations. Patients in the azacitidine + venetoclax arm had higher rates of overall response (although similar complete remission rates) and event-free survival (the primary endpoint). In addition, transplant rates were higher and complication rates lower among patients who received azacitidine + venetoclax. This study, currently only published in abstract form, calls into question the concept that most fit patients should be offered intensive induction therapy. However, further details from this study are needed, including outcomes in specific genetic subgroups, and are awaited eagerly.

Newer molecularly targeted treatments will further add to this challenge. Isocitrate dehydrogenase (*IDH*)-mutated AML is the most common example at present that responds well to less-intensive therapy with newly developed targeted agents. The favorable results obtained with the combination of the *IDH1* inhibitor ivosidenib with azacitidine [5], described in detail in Section 3 (*Frontline Therapy Options for the Unfit Patient*), brings up the question of whether any older patients with this mutation should still be offered intensive chemotherapy. As additional targeted oral therapies such as menin inhibitors become more widely available, it is crucial that the evaluation of fitness be integrated into prospective therapeutic clinical trials to determine if there is differential benefit based on functional characteristics.

### 2.3. Evolving Treatments Require Updated Fitness Assessments

The number of AML treatments—both approved therapies and agents under investigation, singly and in combination—is steadily increasing. The eligibility of patients with AML for intensive chemotherapy is no longer based on a binary distinction between “fit” and “unfit”. With the increasing complexity of AML regimens, both intensive chemotherapy and less-intensive treatments such as HMA + venetoclax now offer clear gradations of treatment intensity. This variability in intensity is illustrated by both standard-of-care and investigational regimens, including the following:

- Fludarabine-based intensification of intensive chemotherapy, using fludarabine, cytarabine and granulocyte colony stimulating factor (G-CSF) ± idarubicin (FLAG or FLAG-Ida) [26];
- The addition of venetoclax [27], gemtuzumab ozogamicin (GO) [28], *IDH1* inhibitors [29], or *FLT3* inhibitors to intensive chemotherapy;
- Triplet HMA-based regimens (NCT06680453; NCT06852222; NCT06652438).

Variability in intensity must be accounted for when considering an individual patient and their disease biology. Patients may also be eligible for multiple treatments of varying intensities at different time points. There is a significant unmet need for tools that assess fitness on a gradient with outputs that help determine the optimal strategy with available treatments.

There is precedent within hematology for assessments of this type. The European Group for Blood and Marrow Transplantation (EBMT) developed tools that describe the intensity of conditioning regimen toxicities in more granular detail, rather than simply a binary distinction between myeloablative and reduced intensity transplant [30,31]. This information is combined with patient fitness and disease-specific information to select an optimal treatment. No such tools exist for AML [8] and there is a need to develop similar instruments for older adults with AML that recognize these nuances.

### 2.4. Dynamics of Fitness During AML Treatment

The 2025 ELN guidelines on fitness assessment strongly endorse the importance of measuring fitness across the entirety of a patient’s treatment course [8]. This “dynamic fitness assessment” informs the modulation of treatment and candidacy for subsequent lines of therapy. A comprehensive fitness assessment prior to the initiation of therapy and with subsequent lines of treatment can guide decision-making and identify vulnerabilities for intervention, as fitness and eligibility for treatment can change over time. Most crucially, we would apply this evaluation to candidacy for alloSCT with a continuous evaluation of eligibility based on performance status.

Dynamic fitness assessment is also highly relevant to patients as many older adults consider functional capacity during treatment an important factor in their treatment decision-making process, especially if treatment is not time-limited [32]. The evaluation of fitness and performance should be performed consistently over time. Importantly, changes in fitness with intensive chemotherapy or less-intensive therapies have prognostic implications for patients who are planning to undergo alloSCT [33]. Patients who may appear unfit at diagnosis, due to AML-related factors, may become more fit for transplant once remission has been achieved; conversely, other patients may become unfit due to treatment-related toxicities.

Work is ongoing in optimizing the dynamics of fitness during treatment. A recent pilot study of physical activity interventions showed that, among newly diagnosed patients receiving intensive chemotherapy, there was a numerically better preservation of physical function in the group randomized to physical activity [34]. CGA-guided fitness optimization strategies have also shown improved results in patients with hematologic malignancies prior to undergoing alloSCT [35]. Fitness optimization should therefore be considered and encouraged along the continuum of care for older adults with AML.

### 2.5. Recommendations: Assessment of Fitness and Suitability for Therapy

1. Treatment decisions regarding intensive vs. less-intensive therapy in older AML patients should be based on assessments of fitness as well as disease biology.
2. Age alone should not be used as an indicator of suitability for different treatment options, although for most older patients, less-intensive combination treatment approaches are preferred.
3. Comprehensive geriatric assessments (CGAs), if available, should be used to better assess overall fitness, identify potential areas of concern and assess for possible early interventions. There are insufficient data to recommend one set of CGA tools over another.
4. Fitness for treatment often changes during the patients’ treatment course and should be re-evaluated on a regular basis.

## 3. Frontline Therapy Options for the Unfit Patient

The median age of onset of AML is approximately 70 years [36]. Most older AML patients, and those with major co-morbidities, are generally considered unfit for standard intensive induction therapy. For such patients, less-intensive treatment approaches should be offered. In the past five years, with the development of newer targeted therapies and combination regimens, there have been major changes in how these patients are treated. This section summarizes the major recent advances in the field and offers consensus recommendations regarding which less-intensive treatments to offer patients. The subsequent section addresses practical issues around how to manage patients undergoing these treatments.

### 3.1. Treatment Options

#### 3.1.1. Monotherapy with HMAs and LDAC

Azacitidine has been compared in two separate randomized clinical trials to conventional care regimens, including LDAC, in newly diagnosed AML patients with 20–30% and >30% bone marrow blasts [37,38]. In aggregate, these studies found that azacitidine was associated with improved overall survival (OS) compared to conventional care regimens, although the OS difference in the AZA-AML-001 study [37] did not reach statistical significance. A subset analysis of the AZA-AML-001 study suggested that patients with adverse risk karyotypes and AML with multilineage dysplasia may derive the greatest benefit with azacitidine vs. conventional care regimens, including LDAC. In contrast, for patients with intermediate risk karyotypes, the outcomes with azacitidine and LDAC were not significantly different.

In terms of choice of HMA, there have been no randomized trials comparing azacitidine to decitabine. However, one study in which physicians were able to select their HMA of choice as standard-of-care vs. guadecitabine found no difference in OS between azacitidine and decitabine [39]. It is likely that these two HMAs are equivalent in overall efficacy.

#### 3.1.2. Venetoclax-Based Regimens

Venetoclax is a selective potent orally available inhibitor of BCL-2, a major anti-apoptotic protein. It has limited single-agent activity in relapsed/refractory (R/R) AML [40], and synergizes with many chemotherapy agents in preclinical AML models. A phase III RCT (VIALE-A) compared azacitidine + venetoclax with azacitidine + placebo, in previously untreated AML patients age 75 and over, or age < 75 with defined co-morbidities precluding intensive chemotherapy [3]. OS was longer and remission rates were higher among patients in the azacitidine + venetoclax arm (Table 1). Responses were also rapid and durable. Nearly half the patients who received azacitidine + venetoclax had a first response (complete remission/complete remission with incomplete hematologic recovery, CR/CRi) before initiation of cycle 2 and a median duration of remission of 17.5 months. Adverse events were more frequent with azacitidine + venetoclax than with azacitidine + placebo, particularly febrile neutropenia (42% vs. 19%, respectively). A subsequent real-world analysis reported comparable results to VIALE-A [41], while others have shown lower median OS of 11–12 months, despite comparable remission rates [42].

Another large phase III RCT (VIALE-C) [4] compared treatment with LDAC + venetoclax with LDAC + placebo, using similar age and co-morbidity criteria to VIALE-A [3]. The difference in OS between the two arms was initially not statistically significant (Table 2) but did become significant with longer follow-up [4,43]. Remission rates were also higher in the LDAC + venetoclax arm [4].

These studies suggest that treatment with venetoclax in combination with either HMAs or LDAC offers benefit over HMAs or LDAC alone, and that the OS benefit is greatest in patients receiving azacitidine + venetoclax [3]. The inclusion criteria for these trials were not identical. VIALE-A specifically excluded patients who had progressed after receiving prior HMA therapy for myelodysplastic syndrome (MDS) [3], while such patients were included in VIALE-C [4]. However, in a subgroup analysis of the latter study, patients who received prior HMA therapy did not appear to demonstrate an OS benefit with LDAC + venetoclax vs. LDAC alone [4,44].

Subsequent studies evaluated prognostic factors in patients treated with azacitidine + venetoclax. Existing ELN risk classification scoring systems, validated for patients receiving intensive chemotherapy [21,45], were found to be poorly discriminative of prognosis in patients receiving azacitidine + venetoclax [46]. Döhner et al. identified a Molecular Prognostic Risk Signature (mPRS) which was highly predictive of OS. Specifically, three distinct prognostic categories were identified: those with lower benefit, consisting of patients with *TP53* mutations; intermediate benefit, including those with *FLT3*-internal tandem duplication (*FLT3*-ITD) and/or *K/NRAS* mutations; and higher benefit, including all other mutational subgroups. The median OS for these groups were 26.5, 12.1 and 5.5 months, respectively [46]. The predictive value of this new molecular scoring system for azacitidine + venetoclax was subsequently validated in real-world cohorts [42,47]. New ELN recommendations for genetic risk classification of adults with AML receiving less-intensive therapies were published in 2024 [48], and are discussed in more detail in the companion article in this issue on “Controversies in the Management of AML in Older Patients”.

A post hoc analysis of patients treated in VIALE-A [3] and in a phase Ib study of azacitidine + venetoclax (NCT02203773) found that patients with *TP53* mutations had a higher CR rate with azacitidine + venetoclax than with azacitidine monotherapy (41% vs. 17%) [49]. However, the OS was not significantly different between the two arms (5.2 vs. 4.9 months, respectively). Patients with adverse risk karyotypes but lacking *TP53* mutations did have an OS benefit with azacitidine + venetoclax, as did other ELN and cytogenetic subgroups.

As mentioned previously, the VIALE-A study excluded patients with prior HMA exposure. Venugopal et al. reported that patients with prior HMA therapy had a 40–50% response rate with azacitidine + venetoclax [50]. In a real-world analysis, Brandwein et al. reported that the OS for patients with prior HMA exposure did not differ significantly from the OS of those without prior exposure [42].

Treatment with azacitidine + venetoclax, or LDAC + venetoclax, requires prophylaxis and monitoring for tumor lysis syndrome (TLS) during the venetoclax ramp-up period, as well as regular complete blood count (CBC) monitoring to assess the need for blood and platelet transfusions and for neutropenia. Please refer to Section 4 (*Protocol-Specific Management*) for details.

A new option for less-intensive therapy is on the horizon, in the form of an all-oral venetoclax and HMA combination. Data presented at the 2025 annual congress of the European Hematology Association (EHA) reported the results of a phase I/II clinical trial evaluating oral decitabine and cedazuridine (DEC-C) combined with venetoclax in patients with newly diagnosed AML [51]. The protocol was similar to the VIALE-A trial, although the sample size in this study was smaller.

The combined phase I and phase IIa/b results demonstrated OS outcomes with venetoclax + DEC-C consistent with what one would expect with venetoclax + azacitidine, with the added advantage of being an entirely oral regimen [51]. The most common grade 3–4 treatment-emergent adverse events, including cytopenias and febrile neutropenia, occurred at rates similar to those observed in VIALE-A. Thirty- and sixty-day mortality rates were low for an older, comorbid patient population. The overall response rate was approximately 63%, comparable to VIALE-A, and patients who achieved CR demonstrated durable responses extending to approximately 12–18 months at the time of reporting [51].

A noteworthy observation relates to venetoclax dosing. In the phase IIb portion, only 50% of patients started on the full 28-day venetoclax schedule. This reflects a trend emerging in clinical practice: early dose modification to mitigate cytopenias while preserving efficacy. DEC-C dosing reductions were modest, with most patients experiencing no more than a one-day reduction even in later cycles [51].

This oral regimen is being submitted as a registrational package to various health authorities and, if approved, will provide the first fully oral less-intensive option for older or unfit AML patients, combining convenience with efficacy comparable to standard venetoclax-based regimens.

#### 3.1.3. Ivosidenib and Azacitidine

IDH1, an enzyme in the citric acid oxidative phosphorylation pathway, is mutated in 6–10% of patients with AML [52]. This mutation results in the production of 2-hydroxyglutarate (2-HG), which functions as an oncoprotein by translocating to the nucleus and blocking the differentiation of myeloid progenitors [53]. Ivosidenib is a potent, selective orally available inhibitor of mutated *IDH1,* which inhibits the production of 2-HG, and restores cell differentiation. Ivosidenib has single-agent activity in relapsed/refractory *IDH1*-mutated AML [52] and is approved for this indication in Canada, the U.S. and Europe.

The phase III randomized AGILE study compared azacitidine + ivosidenib to azacitidine + placebo in patients with previously untreated *IDH1*-mutated AML, ineligible for intensive induction chemotherapy [5]. Azacitidine was administered monthly at standard doses, while ivosidenib was given daily continuously. The azacitidine + ivosidenib arm demonstrated superior outcomes and was well-tolerated (Table 3). The combination of azacitidine + ivosidenib has been approved for untreated, unfit patients with *IDH1*-mutated AML by the U.S. FDA and Health Canada, as well as by other jurisdictions.

#### 3.1.4. Glasdegib and LDAC

A small phase II, randomized, open-label study compared LDAC to LDAC + glasdegib in newly diagnosed patients with AML [55]. OS and CR were higher in the glasdegib arm. Median OS was 8.8 months with LDAC + glasdegib vs. 4.9 months with LDAC monotherapy (HR, 0.5, *p* = 0.0004); CR was 17% vs. 2.3% (*p* < 0.05), respectively. In the phase II BRIGHT AML 1003 trial, patients with secondary AML had a median OS of 9.1 months with LDAC + glasdegib vs. 4.1 months with LDAC alone (HR 0.287, *p* < 0.001) [56]. In patients with de novo AML, median OS was 6.6 months with LDAC + glasdegib vs. 4.3 months with LDAC alone (HR 0.720 (0.395–1.312), *p* = 0.1398). In addition, this study showed a reduction in transfusion requirements with glasdegib, with transfusion independence achieved in 29.3% of patients receiving LDAC + glasdegib vs. 5.6% of patients receiving LDAC alone. Although approved by Health Canada, glasdegib is not publicly funded and is no longer used in Canada by clinicians, having been superseded by the other regimens described.

#### 3.1.5. Duration of Treatment

In the less-intensive therapy studies using HMAs, treatment was usually continued for at least six cycles, unless there was clear evidence of disease progression or intolerance [3,37,38,39]. In the VIALE-A and VIALE-C trials, most responses occurred in the first one to two cycles; however, later responses were observed [3,4]. Nevertheless, most authors felt that if there is no significant reduction in blast percentages or count improvements after two cycles, subsequent remissions are unlikely and treatment changes should be considered. For patients with a targetable mutation, a selective inhibitor can be attempted. For patients with *FLT3*-ITD mutations, switching to the *FLT3* inhibitor gilteritinib [57] or adding gilteritinib to azacitidine + venetoclax [58] would be options.

When a response has been obtained, treatment is generally continued until disease progression or treatment intolerance. However, most patients receiving venetoclax-based combinations will require post-remission dose modifications, as outlined in Section 4 (*Protocol-Specific Patient Management*).

#### 3.1.6. Best Supportive Care (BSC)

All of the aforementioned treatment options are accompanied by supportive care measures, many of which are outlined in Section 4 (*Protocol-Specific Patient Management*). However, for certain patients who have very severe co-morbidities (e.g., prior ECOG PS 4, severe dementia, or debilitating stroke), are pre-terminal, or who decline active treatment after a full discussion of treatment options, BSC alone, +/− hydroxyurea for circulating blast count control, may be a preferred option. This group is not insignificant in number: in a recent retrospective study from Alberta, 36% of newly diagnosed AML patients aged 70–79, and 59% of patients aged 80 and over, received only BSC [59]. BSC management approaches have been reviewed elsewhere [60,61].

#### 3.1.7. Allogeneic Stem Cell Transplantation

Long-term follow-up data from the VIALE A study indicates that most patients will eventually relapse and die, with no evidence to date of a plateau on the OS curve [62]. As azacitidine + venetoclax is therefore considered non-curative therapy, the question arises whether alloSCT can be offered with curative intent. Most older patients are not considered suitable candidates for alloSCT due to advanced age, co-morbidities, and frailty. However, many transplant centers are now offering reduced intensity conditioning (RIC) alloSCT for selected patients up to age 75.

There are data showing that induction with azacitidine + venetoclax followed by alloSCT is feasible in selected patients, with encouraging results [63]. Some patients below age 75 who may have been judged to be unfit for intensive induction therapy based on leukemia-related factors may have an improved performance status after achieving remission, and can then be considered for alloSCT. (For more on alloSCT, see the companion article in this issue on “Controversies in the Management of AML in Older Patients”).

### 3.2. Discussion

Although caution is required when making comparisons across studies, survival outcomes with azacitidine + venetoclax are superior to those with LDAC + venetoclax. The former is therefore the preferred standard-of-care option for most previously untreated AML patients who are unfit for intensive induction therapy.

For patients with *IDH1* mutations, both azacitidine + venetoclax and azacitidine + ivosidenib have been shown to be effective and are acceptable frontline treatment. Again, caution must be exercised when comparing across studies. However, the OS with azacitidine + ivosidenib in the AGILE study appears superior to the OS with azacitidine + venetoclax in the VIALE-A study, even in patients with *IDH1* mutations [3,5]. While there have to date been no head-to-head randomized comparisons of azacitidine + venetoclax vs. azacitidine + ivosidenib, real-world studies also suggest improved outcomes and lower toxicities with azacitidine + ivosidenib in older patients, especially those who are less fit [21,64]. Azacitidine + ivosidenib has an added advantage of producing less myelosuppression in the post-remission period, which can be an ongoing challenge in patients receiving azacitidine + venetoclax (see Section 4, *Protocol-Specific Patient Management*). However, if mutational profiling is not available and the patient needs to begin treatment urgently, or if ivosidenib is not available, azacitidine + venetoclax is acceptable and warranted.

There are several limitations and special considerations. First, azacitidine is administered in a cancer clinic or hospital setting, and some patients may not be able to receive this treatment if they are too frail or lack transportation to reach the clinic setting. LDAC has the advantage of drug stability, allowing for home administration, either alone or in combination with venetoclax or glasdegib. These choices therefore represent reasonable treatment options for patients who are unable to receive azacitidine. However, although these combinations are approved by Health Canada, neither venetoclax nor glasdegib is funded in most of Canada for use with LDAC, limiting their use. Newer oral HMA formulations, including DEC-C and oral azacitidine combined with venetoclax, offer the potential for home administration, but these combinations are not yet approved.

As mentioned, the VIALE-A trial excluded patients who had progressed to AML after receiving prior HMA therapy for MDS. Nevertheless, subsequent studies have indicated that azacitidine + venetoclax can overcome HMA resistance and may induce remissions in many such cases. This regimen is thus acceptable for patients who have previously received HMAs. As an initial alternative, or if azacitidine + venetoclax is ineffective, LDAC + either venetoclax or glasdegib (if available) may be attempted.

As discussed, although patients with *TP53* mutations had a higher CR rate with azacitidine + venetoclax, this did not translate into a survival difference compared with the azacitidine monotherapy arm, due to a very short remission duration. In such patients, it would be reasonable to use azacitidine + venetoclax for induction to optimize the likelihood of achieving remission, and then switch to azacitidine monotherapy for maintenance. This would reduce the subsequent risk of the complications of cytopenias.

### 3.3. Recommendations: Frontline Therapy Options for the Unfit Patient

1. For most newly diagnosed patients not considered candidates for intensive chemotherapy, the standard treatment approach should be the combination of azacitidine + venetoclax.
2. For newly diagnosed patients with *IDH1*-mutated AML, who are not considered candidates for intensive chemotherapy, either azacitidine + ivosidenib or azacitidine + venetoclax would be acceptable as frontline treatment. Although caution should be used in interpreting results across different studies, the combination of azacitidine + ivosidenib would be preferred based on the favorable safety profile and superior overall survival data.
3. In certain instances, other frontline treatments may be used, including LDAC combined with venetoclax or glasdegib, if available. These regimens may be considered if the patient is unable to receive azacitidine, (e.g., due to prohibitive distance from an infusion clinic).
4. In certain instances, azacitidine/decitabine or LDAC monotherapy may be appropriate:
  - Contraindication to, or unavailability of, either venetoclax or ivosidenib;
  - Inability to safely monitor the patient during the induction phase of venetoclax-based therapy;
  - The patient would not be expected to tolerate the myelosuppression associated with azacitidine/LDAC and venetoclax due to significant frailty and poor performance status or due to the presence of significant co-morbidities.
5. Patients with *TP53* mutations may receive either HMA monotherapy or azacitidine + venetoclax. Patients who receive azacitidine + venetoclax and achieve a response may be switched to azacitidine monotherapy as post-remission therapy.
6. For patients who have received prior HMA monotherapy (e.g., for MDS) and have progressed to AML, azacitidine + venetoclax, or LDAC + venetoclax, would be acceptable options, as would azacitidine + ivosidenib for *IDH1*-mutated disease.
7. Enrollment into clinical trials that incorporate novel agents into the azacitidine + venetoclax backbone is strongly encouraged.
8. For patients who decline chemotherapy, or who are not considered candidates for such treatment, or who are pre-terminal, best supportive care alone (+/− hydroxyurea for count control) would be appropriate.
9. In general, less-intensive treatment should be used continuously until disease progression or intolerance, with dose modifications as warranted.
10. Patients with no objective response or evidence of benefit after two or more cycles of azacitidine + venetoclax should be considered for other treatment options, if available.
11. AlloSCT should be considered for highly selected patients in remission with good performance status up to the age of mid 70s; however, most older patients are ineligible.

## 4. Protocol-Specific Patient Management

Supportive care is essential for all AML patients undergoing treatment and should include prophylaxis and the management of TLS, infection, disseminated intravascular coagulation (DIC)/hyperfibrinolysis, bleeding and thrombosis, transfusions, growth factor use, and the management of various therapy-related toxicities [65,66,67]. This section will address supportive care for less-intensive regimens specifically targeting the older unfit AML population.

### 4.1. Management of Patients on Venetoclax-Based Regimens

The addition of venetoclax to either HMA or LDAC adds risks and toxicities which require close monitoring and preventive measures [68]. The incidence of TLS was low in the VIALE-A study (approximately 3%) [3], and slightly higher in VIALE-C [4]. The incidence of febrile neutropenia was significantly higher with azacitidine + venetoclax and LDAC + venetoclax compared with the monotherapy arms. In addition, response assessments are different for azacitidine + venetoclax than for HMA or LDAC monotherapy, which reflect the more rapid responses seen with the combination regimens [3,4].

#### 4.1.1. Inpatient vs. Outpatient Ramp-Up

Although both VIALE-A and VIALE-C required inpatient admission during the 34-day venetoclax ramp-up phases, in practice clinicians have generally been able to safely ramp-up venetoclax on an outpatient basis in the majority of cases [69]. However, some patients should be admitted during the ramp-up phase, including patients with the following:

- Proliferative disease (e.g., markedly elevated lactate dehydrogenase (LDH));
- Evidence of tumor lysis prior to starting therapy (e.g., elevated urate, phosphate);
- Significant baseline renal dysfunction;
- White blood count (WBC) > 20 and unable to adequately cytoreduce with hydroxyurea;
- Inability to monitor adequately as outpatient;
- Hemodynamic instability;
- Coagulopathy (DIC, hyperfibrinolysis);
- Active infection.

#### 4.1.2. TLS Prophylaxis

Measures to reduce the risk of TLS should be applied to all inpatients and outpatients. These include the following:

- Start allopurinol at least 48 h prior to start of venetoclax in all patients unless prior allergy.
- Normalize serum urate, using rasburicase if necessary. X-linked glucose-6-phosphate dehydrogenase (G6PD) deficiency should be ruled out, if possible, prior to giving rasburicase, especially in patients of African, Asian, Mediterranean, or Middle Eastern descent.
- If the patient has signs of acute kidney injury, optimize renal function with hydration prior to start.
- Use intravenous (IV) hydration during ramp-up of venetoclax dosing:
  ○ Inpatient: continuous IV infusion;
  ○ Outpatient: daily IV boluses.
- Closely monitor TLS parameters during ramp-up:
  ○ Inpatient: Monitor potassium, creatinine, phosphate, calcium, uric acid, LDH every 6–8 h in patients with proliferative disease;
  ○ Outpatient: Monitor daily for the first 3–4 days during the ramp-up period.

#### 4.1.3. Venetoclax Dosing

Rapid 3–4 day ramp-up of venetoclax dosing should be used in cycle 1, to minimize the risk of TLS. The standard ramp-up with azacitidine + venetoclax is 100–200–400 mg daily. This should be modified when using azole antifungals (see Table 4).

#### 4.1.4. Venetoclax Duration: Cycle 1

The VIALE-A trial used a venetoclax duration of 28 days [3]. Subsequent studies have shown that the efficacy of shorter venetoclax durations in cycle 1 (7, 14 or 21 days) is comparable to the longer 28-day treatment [70,71], although some studies have indicated that remissions may take longer with shorter durations of venetoclax therapy. Most centers now use 21 days or less in cycle 1, provided blast clearance in the marrow has been documented by day 21. If there is morphologic evidence of leukemia persistence, venetoclax should be continued, and the second cycle started by day 28–30.

#### 4.1.5. Management of Cytopenias During Induction

Blood counts should be monitored at least twice weekly during induction cycles, with transfusion support as per institutional guidelines.

- Antimicrobial prophylaxis should be used during neutropenic phase (absolute neutrophil count (ANC) < 0.5):
  ○ Antibacterial: e.g., levofloxacin (avoid CYP3A inhibitors)
  ○ Antiviral: e.g., acyclovir or valacyclovir
  ○ Antifungal: Incorporate appropriate venetoclax dose adjustments for azoles (Table 4).
- Avoid other CYP3A inhibitors/inducers and P-glycoprotein inhibitors, if possible; incorporate corresponding venetoclax dose modifications if used (Table 4).
- G-CSF (filgrastim) may be used for neutrophil recovery (especially if recovery is delayed), but only after marrow blast clearance has been documented.
- Stop induction cycle venetoclax once marrow blast clearance has been documented.

#### 4.1.6. Response Assessment

A repeat bone marrow aspirate +/− biopsy should be performed prior to the end of cycle 1 (day 21–28, depending on number of days of venetoclax chosen), and then every cycle until clearance of blasts from the marrow. Once remission has been achieved, no further bone marrows are needed unless there is a suspicion of disease recurrence (see Section 4.1.8, *Duration of Therapy*).

#### 4.1.7. Management of Subsequent Treatment Cycles

Regular monitoring of blood counts (at least once every 1–2 weeks) should be continued indefinitely, as cytopenias tend to become more pronounced with increasing number of cycles. Severe neutropenia frequently recurs during subsequent cycles. In order to mitigate the risk, the following interventions are recommended if severe neutropenia (absolute neutrophil count (ANC < 0.5) recurs:

- Stop venetoclax earlier (see below).
- Use G-CSF as needed. In VIALE-A, G-CSF use post-remission was associated with longer duration of remission and reduction in treatment delays, although it is unclear whether these effects were due to the use of G-CSF [72].
- Consider reinstating antimicrobial prophylaxis, e.g., if the patient had prior infections or if the duration of severe neutropenia is expected to be longer than 7 days.
- Most authors do not use more than 21 days of venetoclax per cycle, once remission has been achieved. Although the VIALE-A trial used 28 days for the first cycle, 83% of patients did not receive venetoclax for the full 28 days due to cytopenia. In those who received six cycles or more, the median venetoclax duration was 15–21 days in 69% of patients, and 7% received venetoclax for < 15 days. Real-world studies that adjusted venetoclax scheduling to 14 or 7 days per cycle to limit toxicity showed similar efficacy to VIALE-A [70,71].
- Progressive reductions in the duration of venetoclax treatment to 14, 10 or 7 days per cycle may be needed in subsequent cycles for severe neutropenia and/or thrombocytopenia. If severe cytopenias recur despite these modifications, a reduction in the azacitidine dose (e.g., to 50 mg/m^2^ daily) may be warranted. If azacitidine dose reduction is ineffective, then shortening the duration (e.g., to 5 days) could be considered.
- The daily dose of venetoclax should generally not be reduced, except in the case of drug-drug interactions.
- Subsequent cycles should be delayed (e.g., 1–2 weeks) until adequate count recovery of both neutrophils and platelets. Most patients require 35–42-day cycle lengths, as the neutrophil nadir often occurs in the fourth week.

#### 4.1.8. Venetoclax Duration of Therapy

Treatment cycles should be continued until disease progression or severe intolerance.

Some patients in remission have ongoing severe cytopenias despite maximal reductions in dosing schedules for both venetoclax and azacitidine. In such cases, a repeat bone marrow is indicated to exclude leukemic relapse. Some patients with persistent severe cytopenias will demonstrate underlying dysplastic clones (i.e., MDS) while others have persistently hypocellular marrows despite delays of 6–7 weeks. In such cases, a switch to azacitidine monotherapy may be warranted.

There are situations in which a patient may elect to discontinue treatment (e.g., treatment-related complications, change in goals of care, etc.). Some studies suggest that treatment-free survival can range from 44 to 71 months in those with venetoclax-sensitive mutations, particularly if the patient is measurable residual disease (MRD) negative [73]. However, given the lack of prospective studies in this area, we would not routinely suggest treatment discontinuation, even if MRD negative.

#### 4.1.9. *TP53*-Mutated AML

Discontinuation of venetoclax following marrow blast clearance should be considered in patients with *TP53* mutations who are not earmarked for prompt alloSCT. Most clinicians will continue with azacitidine + venetoclax if the goal is to consolidate for alloSCT. However, in those who are ineligible for transplantation, discontinuation of venetoclax should be considered after a response is obtained, with continuation of azacitidine monotherapy. Venetoclax appears to provide minimal long-term benefit in this patient subset, given the previously discussed data from VIALE [49] and this needs to be weighed against the risk of complications, particularly infection, as *TP53*-mutated AML patients have an inherently higher risk of fungal and bacterial infection [74]. We would suggest enrolling these patients in a clinical trial whenever feasible.

### 4.2. Management of Patients on Ivosidenib-Based Regimens

#### 4.2.1. Dosing

Ivosidenib is given at a dose of 500 mg daily, continuously, during induction and post-remission maintenance. Azacitidine is given at standard doses, i.e., 75 mg/m^2^ daily × 7 day per cycle.

#### 4.2.2. Induction Management

In general, the management of patients undergoing induction therapy with azacitidine + ivosidenib includes a similar use of transfusion support and antimicrobial prophylaxis during the neutropenic phase as described above for venetoclax + azacitidine.

#### 4.2.3. Differentiation Syndrome (DS)

Ivosidenib use may induce DS. In the AGILE study the incidence of DS was 14% in patients receiving azacitidine + ivosidenib and 8% in patients receiving azacitidine + placebo. DS usually occurred in the first month of therapy, but some cases occurred in the second month [5]. Early signs of DS may include any of the following:

- Rising leukocyte count;
- Weight gain and fluid retention;
- Pleural effusions and dyspnea;
- Rising creatinine;
- Fever.

If DS is suspected, therapeutic measures include the use of hydroxyurea for rising leukocyte count, corticosteroids (e.g., dexamethasone 10 mg BID for 3–5 days) and diuretics as needed [5]. In more severe cases, ivosidenib may need to be temporarily held until there is improvement. As signs/symptoms of DS can be non-specific, it may be appropriate to treat for possible infection in parallel with the management of DS.

#### 4.2.4. Electrocardiogram (ECG) Changes

Ivosidenib use may produce QTc prolongation (Table 3). For this reason, other QTc-prolonging drugs should be avoided unless absolutely required. ECGs should be done prior to starting and in the first 2 weeks of treatment. If QTcF (QTc using the Fridericia formula) prolongation to >500 msec occurs, ivosidenib should be held temporarily and any electrolyte abnormalities corrected, until the QTcF improves to <500 msec. If this recurs or persists, the ivosidenib dose may need to be reduced to 250 mg daily.

#### 4.2.5. Post-Remission Monitoring

Weekly blood count monitoring is usually not necessary as ivosidenib does not produce major cytopenias (and rather may result in rapid normalization of neutrophil count during induction). However, azacitidine may cause cytopenias and occasionally renal and hepatic dysfunction, so these need to be monitored at least once prior to each treatment cycle.

#### 4.2.6. Treatment Duration

Ivosidenib treatment should be continued on q4 weekly cycles until disease progression or severe intolerance. Any unexplained major drop in blood counts should prompt a repeat bone marrow to exclude disease relapse/progression.

### 4.3. Management of Patients on Glasdegib-Based Regimens [75]

#### 4.3.1. Dosing and Toxicities

Glasdegib is given at a dose of 100 mg orally daily, in combination with LDAC. In the BRIGHT studies [56,75,76], the major toxicities included alopecia, dysgeusia, fatigue, gastrointestinal toxicities, bone marrow suppression, muscle spasms and musculoskeletal pain, QTc prolongation, rash and weight loss.

#### 4.3.2. Drug Interactions

Glasdegib is metabolized via the CYP3A system. Accordingly, CYP3A inhibitors or inducers may alter serum glasdegib levels and should be avoided if possible. If azole antifungals are used, the glasdegib dose should be reduced to 50 mg daily.

#### 4.3.3. ECG Changes

Glasdegib may produce QTc prolongation. Other QTc-prolonging drugs should be avoided if possible. If concomitant QTc-prolonging agents are used, the QTc interval should be monitored closely. If QTc prolongation to >500 msec occurs, the glasdegib dose should be reduced to 50 mg daily.

#### 4.3.4. Bloodwork

Blood counts, electrolytes, renal and hepatic function should be monitored weekly in the first month of glasdegib use and at least monthly thereafter. Prolonged severe myelosuppression, if not leukemia-related, should prompt discontinuation of glasdegib.

#### 4.3.5. Glasdegib Duration of Therapy

Unless there is clear disease progression, glasdegib treatment should be continued for at least 6 months. Effects on leukemic stem cells may not be observed until after this timeframe.

### 4.4. Repeat Bone Marrow Evaluations and MRD Assessments

When blast clearance has been documented and count recovery has occurred, routine bone marrow aspirates are no longer necessary. However, these should be repeated if there is an unexplained major drop in blood counts beyond the anticipated myelosuppressive effects of treatment, or if circulating blasts reappear and are not G-CSF-related. Repeat marrow assessment should include mutational testing for any potentially targetable mutations.

Evaluation of MRD is now used widely post-treatment in patients receiving intensive chemotherapy, as summarized in the recent 2025 ELN-DAVID guidelines [77]. However, these guidelines do not elaborate on the role of MRD monitoring in patients receiving less-intensive regimens, and routine MRD assessment is not widely used in such patients. Nevertheless, a number of studies have determined that MRD negativity is an important prognostic factor in patients who receive venetoclax-based treatment [78,79,80]. There are selected situations in which this information may be useful, e.g., when stepdown to HMA monotherapy or prolonged interruptions in therapy are contemplated, or in discussing prognosis with patients. A roadmap for the development of MRD testing in AML patients treated with less-intensive regimens has been published by an expert panel from the ELN-DAVID AML MRD working group [81].

### 4.5. Management of Relapsed Disease

Treatment options for relapsed patients treated with azacitidine + venetoclax or other combination regimens are limited:

- If a *FLT3*-ITD or tyrosine kinase domain (TKD) mutation is identified at allelic ratio ≥ 5%, gilteritinib should be used [57].
- If an *IDH1* or *IDH2* mutation is identified, targeted *IDH* inhibitors (ivosidenib or enasidenib, respectively) are approved in the U.S. However, these agents are not currently available in Canada for this indication outside of a clinical trial.
- If an *NPM1* or *KMT2A* mutation is identified, an oral menin inhibitor may be used. Revumenib is available in the U.S. Although no menin inhibitor is currently approved in Canada, many centers have clinical trials with these agents, and patients should be referred to a center offering such studies. Menin inhibitors have induced remission in 25–40% of patients with these mutations [82].
- Other patients should be considered for clinical trials with other investigational agents, if eligible.
- For patients treated with a non-venetoclax-containing regimen, the combination of venetoclax + azacitidine or venetoclax + LDAC may be attempted. Azacitidine + venetoclax has produced remissions in 40–50% of relapsed AML patients [83]. However, venetoclax is not approved for use in Canade in this setting, and there are limited data regarding efficacy following other less-intensive regimens.
- If not eligible for any of these, best supportive care +/− hydroxyurea should be offered. Some patients may be maintained on palliative azacitidine or LDAC monotherapy, in an attempt to delay disease progression.

### 4.6. Summary: Protocol-Specific Patient Management

The optimal management of older patients with AML who are not considered candidates for intensive chemotherapy and who receive less-intensive therapies requires close attention to a number of factors, including appropriate patient selection, early monitoring and prophylaxis for TLS, prevention of cytopenia-related complications and drug-specific toxicities, and appropriate dose modifications. This is particularly important when managing patients on combination regimens and requires considerable expertise. A recent Canadian real-world study found that over time, induction mortality decreased and OS improved [42], underlining the importance of physician experience with these regimens.

## 5. Conclusions and Future Directions

The treatment of AML in older patients has changed significantly since the publication of the first and second Canadian guidelines in 2013 and 2017. The last few years have seen the wide adoption of HMA + venetoclax-based and other less-intensive combinations, together with a decreased emphasis on intensive chemotherapy approaches, although the latter have not disappeared entirely. As a result, AML outcomes in older patients have improved dramatically over the last ten years. In this article, we have addressed practical questions regarding patient selection and management using these regimens, with the goal of providing therapeutic clarity for clinicians. Going forward, it is anticipated that these trends will continue further, with the arrival of newer targeted agents and the wider introduction of venetoclax-based triplet regimens. The availability of these newer regimens will undoubtedly bring new questions and uncertainties that will require future clarification.

## Figures and Tables

**Table 1:** VIALE-A: azacitidine + venetoclax vs. azacitidine + placebo in patients with untreated AML, ≥75 years of age, or <75 years of age with co-morbidities precluding intensive chemotherapy [3].

	AZA + VEN	AZA + PBO	
Median OS	14.1 mos	9.6 mos	HR 0.66 *p* < 0.001
CR	36.7%	17.9%	*p* < 0.001
Median duration of CR	17.5 mos	13.3 mos	
CR/CRi	66.4%	28.3%	*p* < 0.001
By initiation of cycle 2	43.4%	7.6%	*p* < 0.001
Median duration of CR/CRi	17.5 mos	13.4 mos	

AML, acute myeloid leukemia; AZA, azacitidine; CR, complete remission; CRi, complete remission with incomplete hematologic recovery; mos, months; OS, overall survival; PBO, placebo; VEN, venetoclax.

**Table 2:** VIALE-C: LDAC + venetoclax vs. LDAC + placebo in patients with untreated AML ineligible for intensive chemotherapy [4,43,44].

	LDAC + VEN	LDAC + PBO	
Median OS			
Planned primary analysis (median follow-up 12 mos)	7.2 mos	4.1 mos	HR 0.75 *p* = 0.11
Unplanned post hoc analysis (median follow-up 17.5 mos)	8.4 mos	4.1 mos	*p* = 0.04
Remission rates: primary analysis (median follow-up 12 mos)			
CR	27%	7%	*p* < 0.001
CR/CRi	48%	13%	*p* < 0.001
By initiation of cycle 2	34%	3%	*p* < 0.001

AML, acute myeloid leukemia; CR, complete remission; CRi, complete remission with incomplete hematologic recovery; LDAC, low-dose cytarabine; mos, months; OS, overall survival; PBO, placebo; VEN, venetoclax.

**Table 3:** AGILE: Azacitidine + ivosidenib vs. azacitidine + placebo in patients with untreated AML ineligible for intensive chemotherapy [5,54].

	AZA + IVO	AZA + PBO	
Median OS			
Planned primary analysis (median follow-up 12 mos)	24.0 mos	7.9 mos	HR 0.44 *p* = 0.001
Unplanned post hoc analysis (median follow-up 28.6 mos)	29.3 mos	7.9 mos	*p* < 0.0001
CR/CRi	54%	18%	*p* < 0.001
Febrile neutropenia (≥grade 3)	28%	34%	
Neutropenia (≥grade 3)	27%	16%	
Infection (any grade)	28%	49%	
QT prolongation	29%	12%	
DS (any grade)	14%	8%	

AML, acute myeloid leukemia; AZA, azacitidine; CR, complete remission; CRi, complete remission with incomplete hematologic recovery; DS, differentiation syndrome; IVO, ivosidenib; mos, months; OS, overall survival; PBO, placebo.

**Table 4:** Venetoclax dosing schedule for ramp-up phase (3–4 days) in patients with AML, for combination regimens and for concomitant use with azole antifungals.

	Venetoclax Daily Dose (mg)			
	Day 1	Day 2	Day 3	Day 4+
VEN + AZA	100	200	400	400
+ posaconazole	10	20	50	70
+ posaconazole (alternate)	10	20	50	50
+ voriconazole	20	50	70	100
+ fluconazole or isavuconazole	50	100	200	200
VEN + LDAC	100	200	400	600
+ posaconazole	20	50	70	100
+ voriconazole	20	50	70	100
+ fluconazole or isavuconazole	50	100	200	300

AZA, azacitidine; LDAC, low-dose cytarabine; VEN, venetoclax.

## Data Availability

The data discussed in this document are available in the document and the cited references.

## Abbreviations

The following abbreviations are used in this manuscript:

2-HG	2-hydroxyglutarate
alloSCT	allogeneic stem cell transplantation
AML	acute myeloid leukemia
ANC	absolute neutrophil count
BSC	best supportive care
CBC	complete blood count
CGA	comprehensive geriatric assessment
CLSG/GCEL	Canadian Leukemia Study Group/Groupe canadien d’étude sur la leucémie
CR	complete remission
CRi	complete remission with incomplete hematologic recovery
DEC-C	oral decitabine and cedazuridine
DIC	disseminated intravascular coagulation
DS	differentiation syndrome
EBMT	European Group for Blood and Marrow Transplantation
ECG	electrocardiogram
ECOG	Eastern Cooperative Oncology Group
EHA	European Hematology Association
ELN	European LeukemiaNet
FLAG	fludarabine, cytarabine and G-CSF
FLAG-Ida	FLAG and idarubicin
*FLT3*	fms-related tyrosine kinase 3
*FLT3*-ITD	FLT3-internal tandem duplication
G6PD	glucose-6-phosphate dehydrogenase
G-CSF	granulocyte colony stimulating factor (filgrastim)
GO	gemtuzumab ozogamicin
HMA	hypomethylating agent
IDH	isocitrate dehydrogenase
IV	intravenous
LDAC	low-dose cytarabine
LDH	lactate dehydrogenase
MDS	myelodysplastic syndrome
mPRS	Molecular Prognostic Risk Signature
MRD	measurable residual disease
NPM-1	nucleophosmin
OS	overall survival
QTcF	QTc using the Fridericia formula
RCT	randomized controlled trial
RIC	reduced intensity conditioning
R/R	relapsed/refractory
TKD	tyrosine kinase domain
TLS	tumor lysis syndrome
WBC	white blood count
